# Early Impact of Laser Vision Correction (LVC) on the Stability and Quality of the Retinal Image

**DOI:** 10.3390/jcm12051779

**Published:** 2023-02-23

**Authors:** David Smadja, Nir Erdinest, Denise Wajnsztajn, Yishay Weill, Adi Abulafia, David Zadok, Itay Lavy

**Affiliations:** 1 Department of Ophthalmology, Hadassah Medical Center, Faculty of Medicine, Hebrew University of Jerusalem, Jerusalem 9112001, Israel; 2 Department of Ophthalmology, Shaare Zedek Medical Center, Jerusalem 9103102, Israel; 3 Cornea & Refractive Surgery Service, Department of Ophthalmology, Hadassah-Hebrew University Medical Center, Jerusalem 91120, Israel

**Keywords:** laser vision correction (LVC), LASIK, PRK, optical quality, dry eyes, aberrometry, refractive surgery

## Abstract

This retrospective comparative study analyzes the early postoperative impact of laser vision correction for myopia on the optical quality and stability of functional vision using a double-pass aberrometer. Retinal image quality and visual function stability were assessed preoperatively, one and three months after myopic laser in situ keratomileuses (LASIK) and photorefractive keratectomy (PRK) using double-pass aberrometry (HD Analyzer, Visiometrics S.L, Terrassa, Spain). The parameters analyzed included vision break-up time (VBUT), objective scattering index (OSI), modulation transfer function (MTF), and Strehl ratio (SR). The study included 141 eyes of 141 patients, of whom 89 underwent PRK and 52 underwent LASIK. No statistically significant differences were noted between the two techniques in any analyzed parameters at three months postoperatively. However, a significant drop was observed in all parameters one month after PRK. Only the OSI and VBUT remained significantly altered from baseline at the three months follow-up visit, with an increased OSI by 0.14 +/− 0.36 (*p* < 0.01) and a shortened VBUT by 0.57 +/− 2.3 s (*p* < 0.01). No correlation was found between the changes in optical and visual quality parameters and age, ablation depth, or postoperative spherical equivalent. The stability and quality of the retinal images were similar between LASIK and PRK at three months postoperatively. However, significant degradation in all parameters was found one month after PRK.

## 1. Introduction

Dry eyes are one of the most common side effects reported after laser vision correction [1], with approximately 28% of the patients still reporting between mild and severe dry eyes after 3 months [2]. Tear film breakup time and tear production are significantly reduced during the early postoperative course and therefore might affect visual quality [3]. The changes caused by tear film breakup contribute to an increase in optical aberrations and ocular scattering, which leads to a reduction in retinal image quality and correlates with a reduction in visual function [4,5]. Ocular scattering has a significant impact on visual quality [3,6]; however, it has been shown that the use of regular wavefront sensors might overestimate the optical quality by neglecting the contribution of scattering in retinal image quality assessment [7,8]. The association between the objective degradation of the optical quality induced by a refractive procedure and the subjective quality of vision perceived by the patients remains complex, with no clear correlation as one could expect [9,10]. However, in contrast to subjective symptoms reported by the patients, an objective assessment of the optical quality in dry eyes patients, for instance, could help in better quantifying, monitoring and understanding the visual disturbances, often not well characterized by the visual acuity test [6,11]. Objective evaluation of retinal image quality has been successfully reported for monitoring different ocular conditions, such as post-refractive surgery [12], dry eyes [13] and cataract [14], using a double-pass aberrometer method. This technology uses the light emitted on the retina and double passes through the ocular media, after which light reflection is recorded by a charge-coupled camera device. In addition, tear film analysis enables the assessment of dynamic changes in the tear film and its direct impact on retinal image quality. Since tear film changes are dynamic, with evaporation at different speeds between blinks, the observation of real-time changes in the tear film could be a more objective parameter for evaluating dry eye complaints [13].

To the best of our knowledge, the impact of laser vision correction on the stability of functional vision and dynamic changes in optical quality have never been investigated. Therefore, this study aimed to analyze the early postoperative impact of LASIK and PRK on visual and optical quality using real-time observation of retinal image quality with a double-pass aberrometer device.

## 2. Materials and Methods

### 2.1. Ethical Principles

This study followed the tenets of the Helsinki Declaration. This study was approved by the Shaare Zedek Medical Center Institutional Review Board and adhered to the tenets of the Declaration of Helsinki. Shaare Zedek Medical Center Institutional Review Board (IRB) approval (0342-18-SZMC, 19 April 2019) was obtained for this study, and all procedures were carried out per their guidelines.

### 2.2. Study Design

This retrospective comparative study was conducted at the Department of Ophthalmology of Shaare Zedek Medical Center in Jerusalem, Israel.

### 2.3. Study Protocol

The medical files of consecutive patients treated for myopia or myopic astigmatism were reviewed three months postoperatively. The study included 141 right eyes of 141 patients who underwent either femtosecond-assisted LASIK or PRK for myopia and/or myopic astigmatism at the refractive institute of Shaare Zedek Medical Center (Table 1). All patients underwent a complete preoperative ophthalmological examination that included measurements of monocular and binocular uncorrected distance visual acuity (UDVA), corrected distance visual acuity (CDVA), manifest and cycloplegic refraction, slit-lamp biomicroscopy, pupillometry, corneal topography, pachymetry, applanation tonometry, wavefront aberration measurement and dilated fundoscopy. Additionally, optical quality metrics and objective tear film dynamic analysis were assessed preoperatively in all patients using a double-pass aberrometry system (HD AnalyzerTM; 2.7.0.0, Keeler, USA). The following parameters, which are further described below, were analyzed and recorded: objective scatter index (OSI), modulation transfer function (MTF) cutoff frequency, Strehl ratio (SR) and vision break-up time (VBUT). Soft contact lenses and rigid gas-permeable contact lenses were removed at least one or two weeks prior to preoperative examination. Inclusion criteria were all patients considered good candidates for laser vision correction surgery, with myopic spherical equivalent stable over the previous 12 months. Eligible patients for laser vision correction implied the following: normal topography defined as regular and symmetric patterns (including round, oval or symmetric bowtie patterns) or mildly asymmetric patterns (steepening < 0.5D and without a skewed radial axis) based on Placido and Scheimplug-based analysis and stable myopia up to −10 D and/or myopic astigmatism up to −6 D. Photorefractive keratectomy was considered for each patient when the expected preoperative percentage of tissue altered (PTA) exceeded 40% [14], or when it was the preferred choice by the patient after counseling on the risks and benefits of each procedure. Exclusion criteria were: patients below 18 years of age, eyes with previous ocular history, ocular surgery or candidates for hyperopic treatment.

### 2.4. Visual and Optical Quality Assessment

Optical quality metrics of the patients eye and tear film dynamic changes were assessed using a double-pass (DP) aberrometer system and tear film analysis software (HD AnalyzerTM; Visiometrics, Spain) [8]. A 780 nm wavelength laser diode is emitted on the retina and DP through the ocular media, after which a charge-coupled camera device records the light reflection. A personal computer was used to process the retinal images and collect data. The DP images were acquired at best focus and corrected internally by an optometer that ranged from −8.0 to +6.0 diopters (D). Astigmatism was corrected using an appropriate cylindrical lens placed in front of the eye. All measurements were performed by the same experienced technician, starting with the right eye. The objective scatter index (OSI) is a parameter that allows objective evaluation of intraocular scattered light. It was computed by evaluating the amount of light on the periphery (circle of radius between 12 and 20 min of arc) in relation to the amount of light in the central peak of the DP image [15]. Higher intraocular scatter was correlated with higher OSI values. For reference, normal OSI values in a healthy and myopic population have been reported to vary between 0.46 to 1.3 [16,17]. The point spread function (PSF) represents an image projected onto the retina from a point light source. The MTF, directly computed from the PSF, represents the attenuation percentage of the contrast of the retinal image at various resolutions (spatial frequencies), including the combined effects of scattering and high-degree optical aberrations. The value considered in this study was the cutoff point of the MTF curve on the x-axis, which represents the point at which the spatial frequency is at its maximum. The results are presented in cycles per degree. Normal MTF values in a healthy and myopic population have been reported to vary between 53.3 to 31.1 cycles per degree (the higher, the better contrast image quality) [16,17]. The SR is a quantitative measurement of the optical quality of the eye. It can be calculated as the ratio of the peak intensity of the eye’s PSF image from a point source to the maximum attainable intensity using an ideal optical system limited only by diffraction over the system’s aperture [15]. Thus, it is a figure between zero and one. Again, the higher the value, the better the optical quality. As a reference, a normal young eye with a pupil diameter of 4 mm has an SR of approximately 0.3 [7].

The tear film analysis software records dynamic changes in the optical quality through fluctuation of the OSI every 0.5 s while the subject is instructed to avoid blinking. This metric is called the visual break-up time (VBUT) and translates the stability of the visual quality between the two blinks. VBUT is defined as the time between the beginning of the measurement and the time at which the patient’s OSI value deteriorates (increased by 0.5) due to tear film dynamic alterations [13]. Therefore, the VBUT overall duration, which is in other terms the time during which the patient can experience an optimal optical quality vision (preserved point spread function), can be translated into the patient’s “functional visual acuity”. 

### 2.5. Surgical Technique

All surgeries were performed by the same experienced surgeon (DS) under topical anesthesia. In LASIK surgeries, the FS200 femtosecond laser (Wavelight FS200, Alcon Laboratories Inc., Fort Worth, TX, USA) was first used to create a 9 mm diameter and 110 μm thickness corneal flap. Stromal ablations were performed over a 6.5 mm optical zone using the wavefront-optimized ablation profile, with the same excimer laser used in all eyes (Wavelight EX500, Alcon Laboratories Inc., Fort Worth, TX, USA). In all PRK procedures, mitomycin 0.02% solution was used for 30 s after stromal ablation. Then, a balanced salt solution was used for irrigation before a bandage contact lens was placed for one week. Following surgery, all patients were prescribed moxifloxacin 0.5% (q.i.d), dexamethasone 0.1% (b.i.d or q.i.d) for one week as well, and artificial tears (q.i.d) for as long as needed. Steroid treatment was extended and tapered for three months in all patients with PRK. Patients were routinely examined at one day, one week, one month and three months postoperatively, and more if necessary. The one and three months follow-up visits included slit-lamp examination, IOP check, uncorrected distance visual acuity (UCDVA), manifest refraction, corneal topography and retinal image quality using a double-pass aberrometer.

### 2.6. Statistical Analysis

Measurements from the right eye of each patient were used for all analyses to avoid interocular correlations that could bias the analysis. Data were analyzed using Minitab Software version 16 (Minitab Inc., State College, PA, USA). The normality of the data was assessed using the Kolmogorov–Smirnov test. The paired *t*-test was used to compare continuous variables before and after the laser vision correction. The analysis included the Pearson correlation coefficient (r) value, performed using the statistical package for social sciences software 25.0 (SPSS Inc., Chicago, IL, USA). Preliminary sample size calculation was performed and determined as a minimum sample of 32 patients in each group to be enrolled to reach statistically valid conclusions with a 95% confidence level and 80% power.

## 3. Results

**Figure 1:**
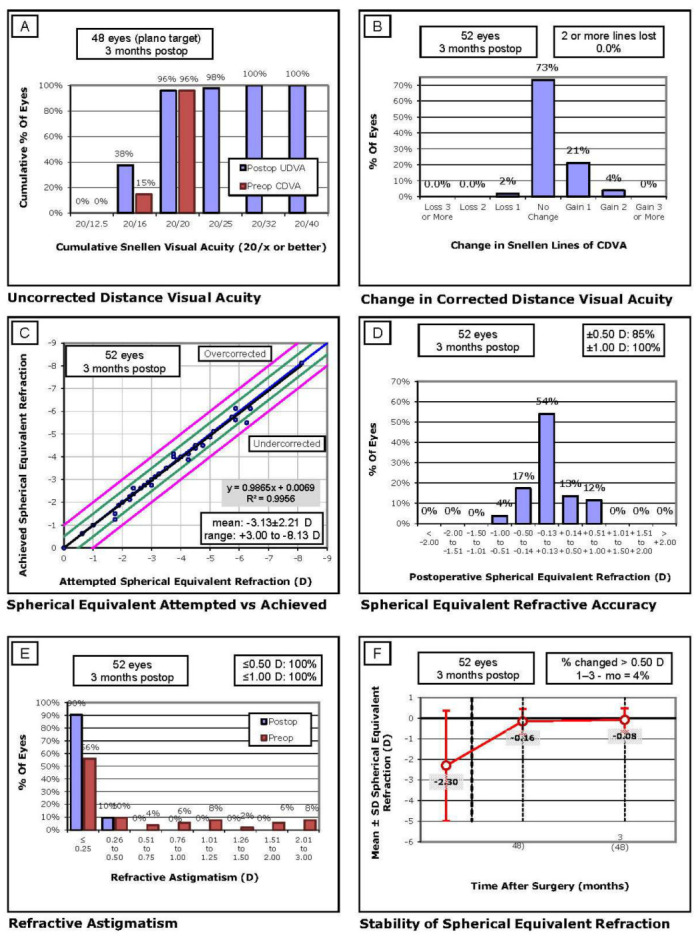
Visual and Refractive Outcomes in the LASIK group. (**A**) Uncorrected distance visual acuity, (**B**) Changes in corrected distance visual acuity, (**C**) Spherical equivalent attempted refraction versus achieved refraction, (**D**) Spherical equivalent refractive accuracy, (**E**) Refractive astigmatism outcomes distribution, (**F**) Stability of spherical equivalent refraction.

**Figure 2:**
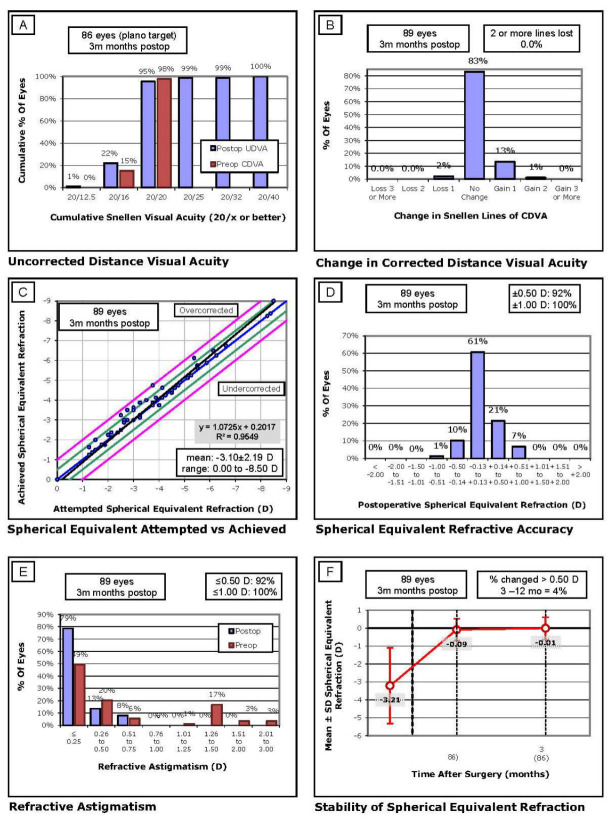
Visual and Refractive Outcomes in the PRK group. (**A**) Uncorrected distance visual acuity, (**B**) Changes in corrected distance visual acuity, (**C**) Spherical equivalent attempted refraction versus achieved refraction, (**D**) Spherical equivalent refractive accuracy, (**E**) Refractive astigmatism outcomes distribution, (**F**) Stability of spherical equivalent refraction.

A total of 141 right eyes of 141 patients who underwent laser refractive surgery, of whom 52 underwent LASIK and 89 underwent PRK, were included in this study (Figure 1 and Figure 2). The baseline clinical and demographic characteristics are summarized in Table 2 and illustrated in Figure 1 (LASIK) and Figure 2 (PRK). No difference was observed at baseline between the two groups, and no statistically significant difference in sphere, cylinder or spherical equivalent was observed between LASIK and PRK at any time point.

### 3.1. Changes in Optical and Visual Quality Metrics

In eyes that underwent LASIK surgery, although a tendency toward mild deterioration in optical and visual quality was observed at one month, no significant difference in any parameters was observed postoperatively. In contrast, in the PRK group, all the parameters analyzed significantly deteriorated at one month, whereas only the OSI and VBUT remained significantly different from baseline at the three months follow-up visit, with an increased OSI by 0.14 +/− 0.36 (*p* < 0.01) and a shortened VBUT by 0.57 +/− 2.3 s (*p* < 0.01). Between one and three months postoperatively, while all the parameters significantly improved in the PRK group, in eyes that underwent LASIK, the only parameter that significantly improved after one month was the VBUT, which increased by 0.78 +/− 1.9 s (*p* = 0.02) between one and three months At three months, no differences in any of the parameters analyzed were found between the two procedures. Postoperative changes in optical and visual quality metrics in both groups are summarized in Table 3, whereas the differences in the changes induced by both procedures are summarized in Table 4.

**Figure 3:**
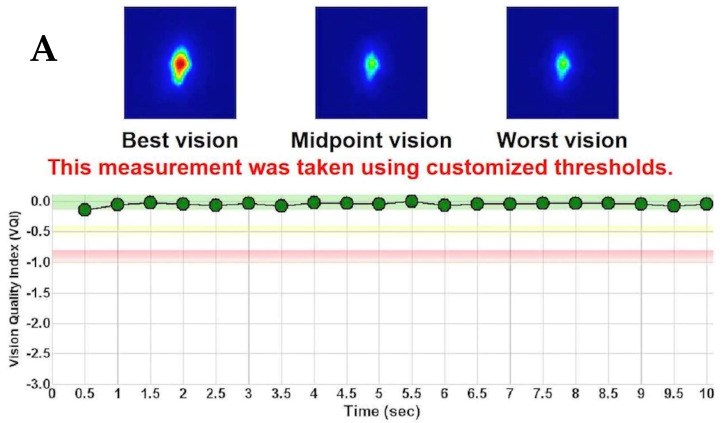

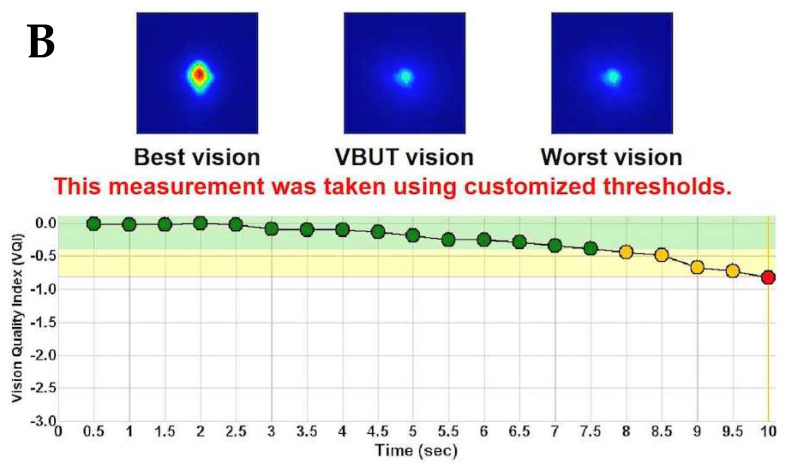
Example of Vision Break-Up Time (VBUT) degradation of a patient 1 month after PRK. (**A**) Stable preoperative vision maintained over 10 s; whereas (**B**) the stability of the retinal image starts to deteriorate after 7.5 s, at the 1-month postoperative visit.

The stability of the retinal image assessed by the VBUT was affected similarly in both groups, although with a more significant drop at 1 month (*p* = 0.04) in the PRK group, before it reached stability similar to that in the LASIK group at 3 months (Figure 3).

The graphic “y-axis” is expressed in Vision Quality Index (VQI), which is a value obtained by inverting and subtracting the OSI value from the best PSF from each of the PSFs obtained during the sequence. Thus, the minimum and best value of the Vision Quality Index is always 0 and would mean that the OSI is not altered at this specific time point of the sequence.

Additionally, the term “customized thresholds” refers to the changes in color bands that appear in the graph and refers to threshold values at which a significant change in OSI is considered: green for an increase between 0 to 0.3, yellow for an increase between 0.31 to 0.5 and red for an increase above 0.5 of OSI.

### 3.2. Correlations

No correlation was found in the LASIK or PRK group between the change in optical and visual quality parameters and the following potential influencing factors: age, ablation depth and postoperative spherical equivalent. However, moderate, but significant, negative correlations were consistently found in both groups, and all the parameters were analyzed between the magnitude of their changes at three months and their preoperative values. All the correlations tested between the parameters are summarized in Table 5 (LASIK) and Table 6 (PRK).

## 4. Discussion

Our findings confirm the relatively low and reversible early optical quality degradation after laser vision correction. Although other metrics have been suggested for assessing the retinal image quality [17,18,19], in this study, we used a double-pass aberrometer system and its derived metrics, which were considered highly reproducible, for analyzing the optical quality of our patients [8]. After an initial drop in all parameters analyzed at one month postoperatively, the optical and visual quality were fully restored at three months after surgery in both procedures, except for the OSI and VBUT, which remained slightly increased (by 0.14) and shortened (by 0.57 s), respectively, in the PRK group. However, although the magnitude of the change that occurred within the first month in the optical quality parameters (OSI, MTF and SR) was more significant in eyes that underwent PRK than LASIK, no difference in optical or visual quality was observed at three months between both procedures. Similar findings were reported when comparing the impact of LASIK and PRK on optical quality metrics, with no significant difference observed between the procedures at three months postoperatively [11,16]. Jung et al. [16] reported a similar postoperative recovery course to that observed in our current study, with optical quality parameters less affected in the LASIK group within the first month and complete recovery in both groups at three months. However, in contrast to the findings reported by Ondategui et al. [11], we found no significant changes in optical quality at three months postoperatively in the LASIK group and only a remaining increase in OSI in the PRK group, whereas the MTF and SR were restored to their preoperative levels. Although the OSI values were similar in both groups, the mean value three months after PRK remained significantly higher than the preoperative levels. Intraocular scattering has been shown to decrease progressively over the first year after PRK [17], and as this study focused on the early postoperative changes up to three months, it is reasonable to presume that this remaining increase in ocular scattering is due to the continuation of the healing process. The epithelium remodeling was reported to persist for at least 6 months, with a gradual hyperplasia over the flattened areas [20]. However, in PRK, the rate of epithelial thickening was found to be superior to other procedures, such as SMILE and LASIK, even after re-establishment of the preoperative epithelial thickness, likely due to the more aggressive wound healing response seen in PRK after the basal membrane disruption [21,22].

However, our more minor postoperative increase in OSI after PRK (by a factor of 1.2) as compared to the study by Ondategui et al. (by a factor of 1.48) might be attributed to the systematic use of mitomycin C 0.02% solution in all our PRK, in contrast to the Ondategui study, where mitomycin was not used. Mitomycin C has proven to be very effective in preventing haze formation by inhibiting the replication of myofibroblast progenitor cells in the anterior stroma, which usually occurs naturally following the normal keratocyte apoptosis response to epithelial removal [18]. Additionally, modern excimer lasers and ablation profiles have considerably improved the regularity and smoothness of the stromal bed after ablation, which led to a lower risk of haze development postoperatively [23,24].

The VBUT parameter, which assesses the retinal image stability between two blinks, deserves a separate analysis by novelty. The term “break-up time”, associated with this parameter, represents the time it takes between two blinks to lose functional vision due to an unstable tear film. In this study, we found that the VBUT was mainly affected during the first month after surgery, with a shortening of 0.64 s in the LASIK group (*p* = 0.09) and 1.63 s in the PRK group (*p* < 0.01). However, in both procedures, the duration of the VBUT was statistically significantly improved between 1 and 3 months, with no difference in the VBUT values observed at 3 months between LASIK and PRK (*p* = 0.11), with 9.6 ± 1.05 s and 9.1 ± 2.2 s, respectively. A stable tear film over a sufficiently long period is crucial for benefiting from proper sight. In the early postoperative course after laser vision correction, patients often report visual disturbances and fluctuations throughout the day, despite good uncorrected visual acuity. In Figure 1, we can see the postoperative shortened VBUT of a patient 1 month after LASIK, with a 20/20 uncorrected vision, but a functional vision maintained for only the first 5.5 s, until it deteriorates quickly. It has been shown that patients with a short tear film BUT (TBUT) may show completely normal visual acuity because of the ability of the patient to read clearly right after the eye opened [19]. As shown in Figure 1, although the tear film might be unstable, it remains smooth enough for this short period to provide functional vision and enable the patient to read the 20/20 line on the visual chart successfully. However, patients with unstable tear films complained more of eyestrain and blurry vision than aqueous-deficient dry or healthy eyes [25]. The early postoperative alteration of functional visual acuity after LASIK and PRK, as reported in our study with the reduction in VBUT within the first month, helps better understand the discrepancy that often arises between the good visual acuity achieved and subjective patient complaints. Although not in the scope of this current study, a prospective analysis that would properly correlate the patient’s early postoperative subjective visual complaints with the objective measurement of the visual BUT temporal changes could further validate this hypothesis and emphasize the use of such a metric in the future.

Using another technology developed to continuously assess visual acuity over one minute continuously, Kaibo et al. [20,26] also reported the prevalence of reduced functional visual acuity in patients with short TBUT dry eye. The frequent association between reduced goblet cell density and short BUT was initially reported by Toda et al. [21]. More recently, Den et al. [25] and Uchino et al. [22] suggested the possible involvement of mucin components as significant players in short TBUT development. After laser refractive surgery, a decrease in conjunctival goblet cell population in the early postoperative period in both procedures [27], LASIK and PRK, and a temporary reduction in tear film break-up time was reported [23,27]. However, this is the first time that the VBUT, which directly assesses the visual impact of the unstable tear film induced by surgery, was analyzed after laser vision correction. In a previous study from our group, we showed the negative impact in healthy patients of a short reading session on a smartphone screen on visual quality stability, with a reduction in VBUT and deterioration of the retinal image quality [24]. Dynamic optical quality changes using VBUT metrics have also been reported in dry eye patients [12] in an attempt to explain and reduce the discrepancy often found between the standard clinical test in use for evaluating dry eye patients and subjective symptoms reported [28,29]. 

Another interesting finding from this current study is the negative correlations between the magnitude of the degradation in optical quality and the optical quality preoperative level. Although it would need to be further confirmed in larger sample studies, it seems that the higher the preoperative optical quality measured was, the more tendency there was to record a transient reduction postoperatively. However, it seems understandable that the better the optical quality preoperatively, the higher the risk of experiencing a transient decrease after a laser procedure. For a better understanding, it could be compared to a professional athlete who would have a greater chance to lose his first place every time he is competing, because there is no better ranking rather than because any other athlete that is lower ranked could improve. This relationship was found for every metric measured in this study, except for the lack of association between the VBUT changes and the other optical quality metrics, such as OSI, MTF and SR. Indeed, the VBUT degradation is related to a temporal change due to the tear film alteration that occurs between two blinks. It does not clinically reflect the optical quality magnitude of any of the parameters analyzed, but rather the duration of the retinal image quality (point spread function) maintenance between two blinks.

In conclusion, LASIK and PRK temporarily affected the optical quality of the eye at the early postoperative stage, with an increased scattering (OSI) and reduced contrast vision (MTF and SR). Although observed in both procedures, a more significant deterioration in all optical quality parameters was noted in the PRK group after 1 month, but with restoration of the optical quality by 3 months postoperatively. The immediate, although temporary, postoperative shortening in the vision break up time (VBUT) in both procedures, which is translated by a limited maintenance of optical quality between two blinks, is reported for the first time in this study, and it might contribute to a better understanding of the discrepancy that could often be observed between early postoperative patient complaints of visual fluctuations and the good visual acuity findings obtained by standard visual and clinical assessment methods [11].

## Figures and Tables

**Table 1:** Baseline Clinical and Demographic Characteristics (Mean ± SD; range). SD = Standard Deviation; SE = Spherical Equivalent; D = Diopters; OSI = Objective Scatter Index; MTF = Modulation Transfer Function; c/deg = cycle per degree; SR = Strehl Ratio; VBUT = Visual Break Up Time; Sec = second.

Parameters	LASIK (n = 52 Eyes)	PRK (n = 89 Eyes)	*p*
Age (years)	36.17 ± 10.3 (22; 57)	33.9 ± 10.1 (18; 55)	0.21
Gender (% female)	49% (n = 26)	51.6% (n = 48)	0.08
Preop SE (D)	−2.3 ± 2.67 (−8; −0.75)	−3.21 ± 2.12 (−8.5; −1.25)	0.05
Postop SE (D)	−0.08 ± 0.57 (−1; 0.5)	−0.01 ± 0.62 (−0.5; 1)	0.54
OSI	0.65 ± 0.40 (0.2; 1.8)	0.66 ± 0.32 (0.1; 2.1)	0.87
MTF (c/deg)	39.5 ± 8.5 (18.7; 53.9)	38.2 ± 8.9 (17.3; 54.1)	0.65
Strehl Ratio	0.22 ± 0.05 (0.12; 0.33)	0.21 ± 0.05 (0.11; 0.34)	0.15
Visual BUT (sec)	9.47 ± 1.3 (5; 10)	9.7 ± 1.1 (3; 10)	0.09

**Table 2:** Postoperative Refractive Outcomes (mean ± SD; range) spherical equivalent; D = diopters; SD = standard deviations.

Parameters	LASIK (n = 52)	PRK (n =89)	*p*
Postop 1 Month			
Sphere (D)	−0.08 ± 0.6 (−1.25; 0.75)	0.12 ± 0.58 (−0.75; 1.5)	0.14
Cylinder (D)	−0.39 ± 0.13 (−0.5; 0)	−0.62 ± 0.33 (−1.5; −0.25)	0.08
SE (D)	−0.16 ± 0.61 (−1.25; −0.5)	−0.09 ± 0.61 (−1; 1.25)	0.96
**Postop 3 Months**			
Sphere (D)	−0.07 ± 0.6 (−1; 0.75)	0.17 ± 0.66 (−0.5; 2.5)	0.17
Cylinder (D)	−0.35 ± 0.24 (−0.5; 0)	−0.49 ± 0.18 (−0.75; −0.25)	0.07
SE (D)	−0.08 ± 0.57 (−1; 0.5)	−0.01 ± 0.62 (−0.5; 1)	0.54

**Table 3:** Differences in optical quality metrics and visual stability function at different time points between LASIK and PRK (mean ± SD). OSI = Objective Scatter Index; MTF = Modulation Transfer Function; c/deg = cycle per degree; SR = Strehl Ratio; VBUT = Visual Break Up Time; *p* in bold = change that is statistically significant.

Baseline	LASIK (n = 52)	PRK (n = 89)	*p*
OSI	0.65 ± 0.40	0.66 ± 0.32	0.87
MTF (c/deg)	39.5 ± 8.5	38.2 ± 8.9	0.65
Strehl Ratio	0.22 ± 0.05	0.21 ± 0.05	0.15
Visual BUT (sec)	9.47 ± 1.3	9.7 ± 1.1	0.09
**Postop 1M**			
OSI	0.78 ± 0.54	1.2 ± 0.67	**<0.001**
MTF (c/deg)	38.8 ± 10	30.9 ± 10.1	**<0.001**
SR	0.22 ± 0.05	0.16 ± 0.06	**<0.001**
VBUT (sec)	8.8 ± 2.1	8.1 ± 3.2	0.17
**Postop 3M**			
OSI	0.75 ± 0.43	0.8 ± 0.36	0.45
MTF (c/deg)	38.3 ± 10.1	36.2 ± 9.1	0.22
SR	0.21 ± 0.04	0.2 ± 0.04	0.11
VBUT (sec)	9.6 ± 1.05	9.1 ± 2.2	0.11

**Table 4:** Differences in the magnitude of the changes in optical quality metrics induced by LASIK and PRK at different time points (mean ± SD, *p*). Δ1M = Difference at 1month from Baseline; Δ3M = Difference at 3 months from Baseline; Δ1–3M = Difference between 1 and 3 months; OSI = Objective Scatter Index; MTF = Modulation Transfer Function; c/deg = cycle per degree; SR = Strehl Ratio; VBUT = Visual Break Up Time; *p* in bold = change that is statistically significant.

	LASIK (n = 52)	PRK (n = 89)	*p* (LASIK vs. PRK)
**Δ1M**			
OSI	0.14 ± 0.35 (0.18)	0.54 ± 0.58 **(<0.01)**	**<0.001**
MTF (c/deg)	−1.24 ± 8.3 (0.7)	−7.7 ± 11.6 **(<0.01)**	**0.002**
Strehl Ratio	−0.01 ± 0.05 (0.89)	−0.04 ± 0.06 **(<0.01)**	**0.001**
Visual BUT (sec)	−0.64 ± 2.1 (0.09)	−1.63 ± 3.1 **(<0.01)**	**0.04**
**Δ1–3M**			
OSI	−0.04 ± 0.24 (0.77)	−0.42 ± 0.56 **(<0.01)**	**<0.001**
MTF (c/deg)	0.09 ± 7.9 (0.82)	6.24 ± 10.1 **(<0.01)**	**<0.001**
SR	−0.01 ± 0.04 (0.57)	0.04 ± 0.06 **(<0.01)**	**<0.001**
VBUT (sec)	0.78 ± 1.9 **(0.02)**	1.1 ± 2.7 **(0.01)**	0.54
**Δ3M**			
OSI	0.10 ± 0.31 (0.23)	0.14 ± 0.36 **(<0.01)**	0.45
MTF (c/deg)	−1.17 ± 10 (0.5)	−1.93 ± 11 (0.15)	0.68
SR	−0.01 ± 0.06 (0.46)	−0.01 ± 0.05 (0.57)	0.73
VBUT (sec)	0.15 ± 1.9 (0.53)	−0.57 ± 2.3 **(0.01)**	0.06

**Table 5:** Correlations between the changes in visual and optical quality metrics 3 months after LASIK with possible influencing factors. SE = Spherical Equivalent; OSI = Objective Scatter Index; MTF = Modulation Transfer Function; SR = Strehl Ratio; VBUT = Vision Break Up Time; r = Pearson correlation coefficient; Bolded values represent statistically significant correlations with *p* < 0.05.

r Coefficient (*p* Value)	Age	Ablation Depth	Postop SE	Preop OSI	Preop MTF	Preop SR	Preop VBUT
**ΔVBUT**	−0.04 (0.97)	−0.11 (0.48)	−0.25 (0.07)	0.05 (0.78)	0.02 (0.9)	0.02 (0.9)	−0.71 **(*p* < 0.01)**
**ΔOSI**	0.19 (0.17)	−0.03 (0.8)	−0.05 (0.78)	−0.28 **(0.03)**	0.31 **(0.02)**	0.34 **(0.02)**	0.19 (0.17)
**ΔMTF**	−0.15 (0.28)	0.08 (0.57)	0.01 (0.95)	0.25 **(0.04)**	−0.41 **(*p* < 0.01)**	−0.44 **(*p* < 0.01)**	−0.14 (0.32)
**ΔSR**	−0.21 (0.06)	0.09 (0.53)	−0.25 (0.07)	0.26 **(0.04)**	−0.31 **(0.02)**	−0.63 **(*p* < 0.01)**	−0.2 (0.15)

**Table 6:** Correlations between the changes in visual and optical quality metrics 3 months after PRK with possible influencing factors. SE = Spherical Equivalent; OSI = Objective Scatter Index; MTF = Modulation Transfer Function; SR = Strehl Ratio; VBUT = Vision Break Up Time; r = Pearson correlation coefficient; Bolded values represent statistically significant correlations with *p* < 0.05.

r Coefficient (*p* Value)	Age	Ablation Depth	Postop SE	Preop OSI	Preop MTF	Preop SR	Preop VBUT
**ΔVBUT**	−0.03 (0.97)	−0.01 (0.9)	0.14 (0.19)	0.03 (0.78)	0.09 (0.38)	0.06 (0.64)	−0.55 **(*p* < 0.01)**
**ΔOSI**	0.01 (0.9)	0.01 (0.9)	0.08 (0.78)	−0.44 **(*p* < 0.01)**	0.46 **(*p* < 0.01)**	0.28 **(0.01)**	0.19 (0.08)
**ΔMTF**	0.03 (0.78)	0.01 (0.9)	−0.01 (0.91)	0.27 **(0.01)**	−0.59 **(*p* < 0.01)**	−0.35 **(*p* < 0.01)**	−0.13 (0.22)
**ΔSR**	0.1 (0.35)	−0.02 (0.8)	0.01 (0.97)	0.31 **(0.01)**	−0.59 **(*p* < 0.01)**	−0.57 **(*p* < 0.01)**	0.1 (0.5)

## Data Availability

The datasets that support the findings of our study are available upon reasonable request from the corresponding author (D.S.); however, prior approval of proposals must be applied for from our institution’s data security management, and a signed data-sharing agreement will then be approved.
